# Expression of Merkelcell polyomavirus (MCPyV) large T-antigen in Merkel cell carcinoma lymph node metastases predicts poor outcome

**DOI:** 10.1371/journal.pone.0180426

**Published:** 2017-08-01

**Authors:** Georg Haymerle, Stefan Janik, Alexandra Fochtmann, Johannes Pammer, Helga Schachner, Lucas Nemec, Michael Mildner, Roland Houben, Matthaeus Ch. Grasl, Boban M. Erovic

**Affiliations:** 1 Department of Otolaryngology Head and Neck Surgery, Medical University of Vienna, Vienna, Austria; 2 Department of Surgery, Clinical Division of Plastic and Reconstructive Surgery, Medical University of Vienna, Vienna, Austria; 3 Department of Clinical Pathology, Medical University of Vienna, Vienna, Austria; 4 Department of Dermatology, Research Division of Biology and Pathobiology of the Skin, Medical University of Vienna, Vienna, Austria; 5 Department of Dermatology, Medical University of Wuerzburg, Wuerzburg, Germany; Fu Jen Catholic University, TAIWAN

## Abstract

**Background:**

The aim of this study was to determine the prevalence of MCPyV in Merkel cell carcinoma (MCC) primaries versus lymph node metastasis and to evaluate possible prognostic factors.

**Methods:**

Samples of MCC primaries and lymph node metastases were stained immunohistochemically for the MCPyV large T-antigen and expression was compared to patients´ clinical outcome.

**Results:**

41 MCC patients were included. 33 (61%) out of 54 specimens were MCPyV-positive in the immunohistochemistry. 15 (47%) out of 32 primary tumors were positive compared to 18 (82%) out of 22 lymph node metastases. Eleven patients with positive polyomavirus expression died from the carcinoma compared to 4 patients without virus expression. Cox regression analysis showed worse disease-free survival in patients with MCPyV compared to virus-negative lymph nodes (p = 0.002).

**Conclusions:**

To our knowledge this is the first study to describe a negative prognostic effect of the MCPyV expression in lymph node metastasis in MCC patients.

## Introduction

Merkel cell carcinoma (MCC) is the most aggressive skin cancer and found as neuroendocrine neoplasm predominantly in elderly white patients [1]. The incidence of 1500 new cases annually in the United States is very low compared to other cutaneous malignancies reported as and depicts a challenging disease regarding diagnosis and appropriate treatment [2,3]. MCC is typically presented as a flat or raised, isolated, red-purplish lesion with a shiny surface [4].

In 2008 the Merkelcell polyomavirus (MCPyV) has been found to be associated with the pathogenesis in MCC patients. Feng and colleagues hypothesized that the viral DNA is integrated into the tumor genome [5]. In the current literature 11 human polyomaviruses have been described. However, the MCPyV is the only polyomavirus associated with carcinogenesis in the human. The virus seems to effect classical carcinoma hallmarks including inhibition of tumor promoters, prevention of apoptosis and stimulation of tumor angiogenesis [6]. Nevertheless, the MCPyV large T-antigen (LTA) expression in MCC tumors varies between 40 to 100% in current studies and has been shown to be higher in skin areas less exposed to UV light. Hence the general pathogenesis and its role in early lymph node metastases in particular remain to be elucidated. We therefore conducted a retrospective chart review of MCC patients and compared MCPyV LTA expression in the tumor of the skin with regional lymph node metastases. Expression levels and pathologic as well as clinical data were correlated.

## Material and methods

### Patients

The institutional research ethics board approved the study and waived the need for written informed consent for obtaining medical records (REB No. 1798/2013). A single-institution retrospective medical chart review was performed of all MCC patients treated at the Medical University of Vienna between 1994 and 2015. Cases with unavailable histological tissue samples were excluded.

Demographic, clinical and pathological data were retrieved from hospital records, thus including sex, age, tumor localization, TNM classification, and management of the primary tumor, follow-up, and cause of death. Throughout the study, therapy included surgery followed by radiotherapy in patients with lymph node metastasis or positive resection margins. Chemotherapy was given in case of distant metastases. Recurrence rate, follow-up and outcome were evaluated. Duration of follow-up was calculated from the date of first diagnosis to the date of death or last follow-up.

The clinical staging system was used according to the American Joint Committee on Cancer (AJCC) [7]. Demographic and pathologic data were summarized using descriptive statistics. Continuous data was described with mean, median, minimum and maximum values.

### Tissue samples

Formalin-fixed, paraffin-embedded tissue samples of patients treated for MCC were obtained from the Clinical Department of Pathology and the Department of Dermatology at the Medical University of Vienna. In total 54 samples from primary tumors or nodal metastases were collected. The missing tissue samples were not retrievable from the institutions’ archives. Histological diagnosis was confirmed by the Departments of Clinical Pathology or Department of Dermatopathology. MCC specific staining included cytokeratin 20 (CK20), neuron-specific enolase (NSE), chromogranin A (CrA), neural cell adhesion molecule (NCAM) and thyroid transcription factor-1 (TTF-1).

### Immunohistochemistry

Samples were immunostained for MCPyV LTA with the antibody CM2B4 (sc136172, 1:200; Santa Cruz, CA, USA) as described before [8]. Dewaxed and rehydrated tumor paraffin sections (of 2–3 μm thickness) were subjected to heat retrieval. For MCPyV LTA antibody heat retrieval was conducted via autoclave (1 bar) using citrate buffer (pH 6.0). Sections were detected with UltraVision LP Detection System HRP Polymer (Thermo Fisher Scientific Inc.). After blocking of endogen peroxidase (3% H2O2 in PBS), slides were exposed to Ultra V Block for 5 min. The primary antibody (diluted in 1% BSA) was incubated 60 min at room temperature in a humidity chamber. Following three washing steps in PBS, the Primary Antibody Enhancer was applied for 10 min and after further three times washing in PBS, the HRP-Polymer for 15 min. The Signal was detected with Metal Enhanced DAB Substrate, stained for 2min. Counterstaining was performed with Mayer's hemalum. Negative control was done without primary antibody.

### Histological analysis

**Fig 1:**
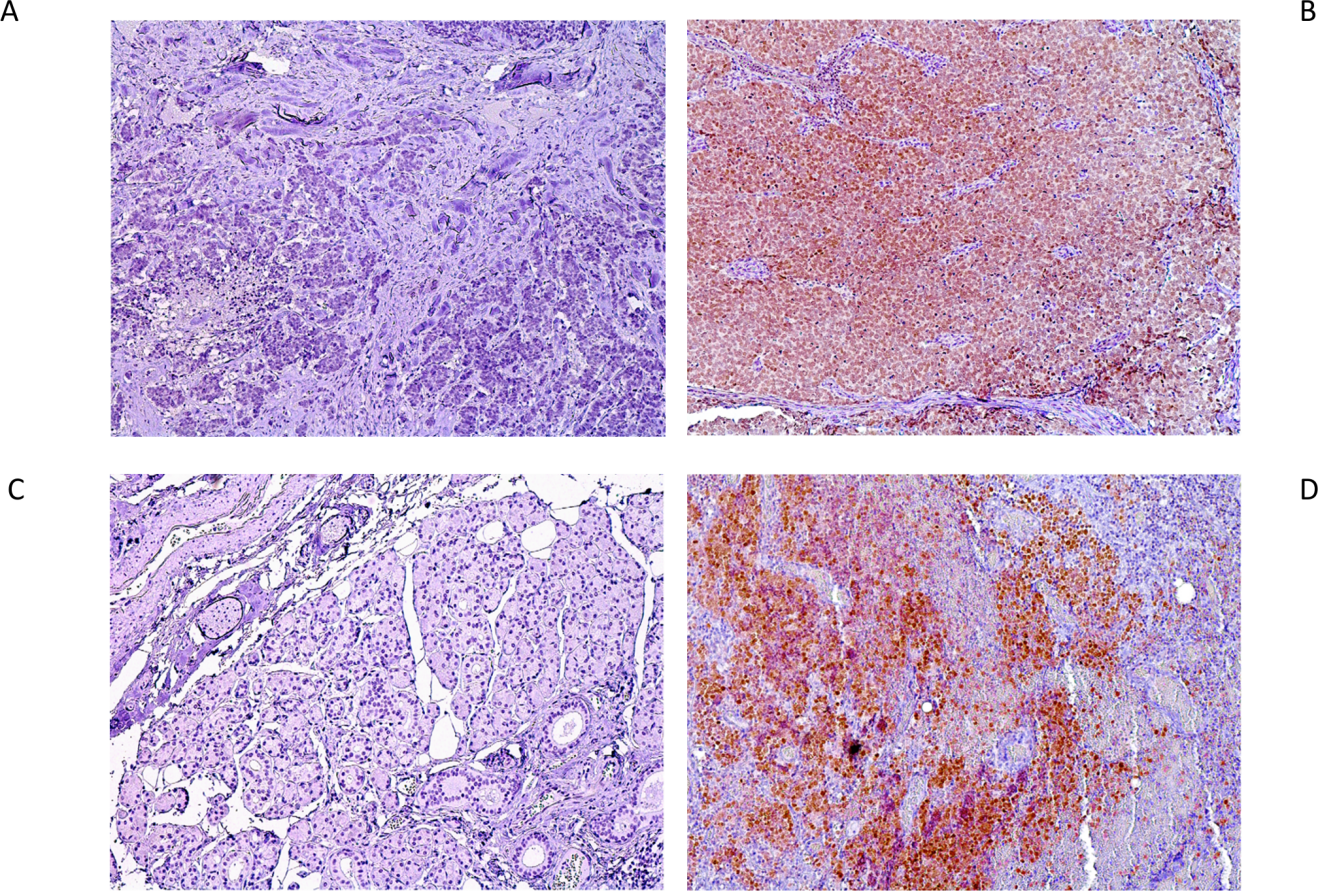
Immunohistochemistry of the MCPyV large T-antigen (LTA) shows that the antigen is predominantly expressed in the nuclei of Merkel cell carcinoma cells. Panel (A) demonstrates no expression of the primary tumor whereas in panel (B) a positive expression of MCPyV LTA can be appreciated. In Panel (C) one can observe no LTA expression compared to positive expression in lymph node metastases (D). All photomicrographs are taken at x100 magnification.

The expression of the MCPyV LTA in the tumor tissue was determined independently by two investigators (JP and GH). The tumor cell expression of MCPyV was quantified as a percentage of the total number of tumor cells and the proportions were assigned to 1 of 3 categories: <10% = negative; 10% to 60% = moderate; and 61% to 100% = strong. Positive (moderate and strong) and negative staining of primary tumor and lymph node metastases are shown in Fig 1 A-1D. An observer bias was minimized by repeating the evaluation of protein expression at two different time points and without knowledge of patients’ clinical data. Results were correlated with the sociodemographical, clinical and pathological data of all patients.

### DNA extraction and MCPyV detection by PCR

**Fig 2:**
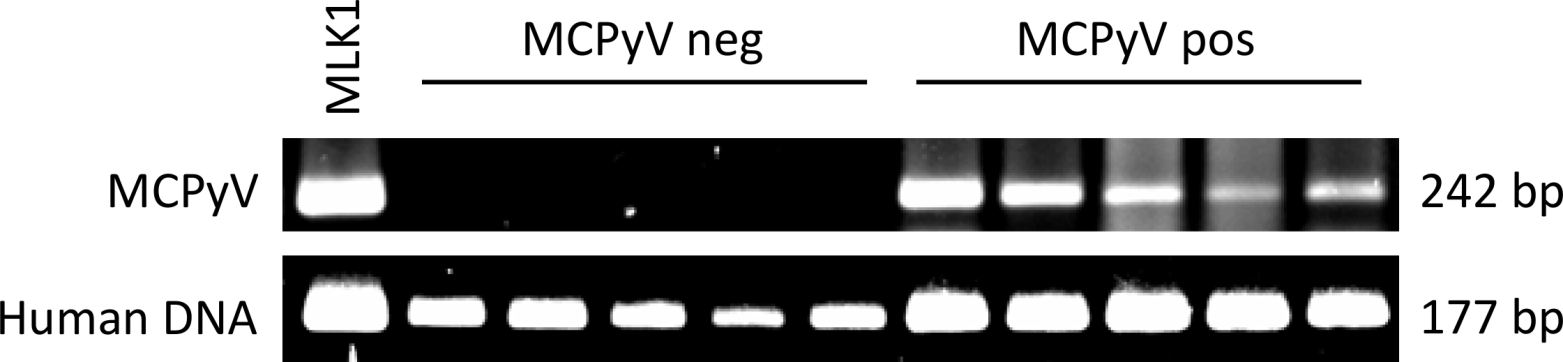
Five samples, which were negative and 5 samples, which showed positive immunostaining, were used for PCR. The MLK1 Merkel cell carcinoma cell line was used as positive control for the establishment of the MCPyV PCR (Fig 2).

DNA from paraffin embedded MC samples was prepared using the QIAamp DNA FFPE Tissue Kit (Qiagen, Hilden, Germany) according to the manufacturer’s instructions. Three 5μm sections were used from each sample. Five samples, which were negative and 5 samples, which showed positive immunostaining were used for PCR. The MLK1 Merkel cell carcinoma cell line was used as positive control for the establishment of the MCPyV PCR (Fig 2). Conventional PCRs were performed in a volume of 50 μL containing 0.25 μL peqGOLD Taq-DNA-Polymerase (PEQLAB, Erlangen, Germany), 5 μL 10× reaction buffer Y (PEQLAB), 10 μl 5x enhancer solution (PEQLAB), 1μL dNTPs (25 mM), 1.5 μL of each primer (10 μM), and 30.75 μL H_2_O. The following forward and reverse primers were synthesized by VBC Genomics (VBC Genomics, Vienna, Austria): for MCPyV (forward: 5′-GATCAGGAGGATTCAGCTTCG-3′, reverse: 5′-CAGAGGATGAGGTGGGTTCC-3′) and for genomic DNA (Loricrin; forward: 5′-GGAGTTGGAGGTGTTTTCCA-3′, reverse: 5′-ACTGGGGTTGGGAGGTAGTT-3′). The PCR included 2 min at 95°C for initial denaturing, followed by 35 cycles of 15 s at 95°C, 15 s at 55°C, 50 s at 72°C, and a final extension step at 72°C for 10 min. Amplicons were subjected to electrophoresis in a 1.0% Agarose gel containing GelRed™ (Biotium, Fremton, CA). The specificity of the PCR products was confirmed by sequencing (not shown).

### Statistical analysis

Statistical analysis was performed using the Statistical Package for the Social Sciences software (SPSS version 15.0). Sociodemographic and pathologic data was summarized using descriptive statistics (Tables 1 and 2). Recurrence and survival rates were calculated from the date of first diagnosis to the event of interest. Recurrences were classified as local, regional, or distant.

**Table 1:** MCPyV status and clinicopathologic data of 41 patients with Merkel cell carcinoma.

	MCPyV positive (%)*	MCPyV negative (%)*	Total (%)*
**Sex**			
Female	11 (61)	7 (39)	18
Male	15 (65)	8 (35)	23
**Age**			
Median (Range)	74 (53–85)	79 (46–93)	76 (46–93)
**Staging**			
I	10 (24)	9 (22)	19 (46)
II	3 (7)	3 (7)	6 (15)
III	8 (20)	2 (5)	10 (24)
IV	1 (2)	1 (2)	2 (5)
MCCUP	4 (10)	0	4 (10)
**Localization**			
Head and Neck	9 (43)	12 (57)	21 (51)
Extremities	10 (100)	0	10 (24)
Trunk	3 (50)	3 (50)	6 (15)
No primary	4 (100)	0	4 (10)
**Recurrent disease**			
Yes	15 (71)	6 (29)	21 (51)
No	11 (55)	9 (45)	20 (49)

Abbreviations: MCPyV, Merkel cell polyomavirus; MCCUP, Merkel cell carcinoma of unknown primary.

* Values represent number of patients (%) except as stated otherwise.

**Table 2:** Immunohistochemistry of MCPyV large T-antigen (LTA) and outcome data in 54 specimens from 41 patients with Merkel cell carcinoma.

MCPyV status	Total (%)	Primary tumor	Lymph nodes	ANED	AWD	DNED	DOD
Positive	33 (61)	15 (47)	18 (82)	8 (62)	1 (100)	6 (50)	11 (73)
Negative	21 (39)	17 (53)	4 (18)	5 (38)	0 (0)	6 (50)	4 (27)
N/A	14	9	5				
Total	54	32	22	13	1	12	15

Abbreviations: MCPyV, Merkel cell polyomavirus; ANED, Alive, no evidence of disease; AWD, alive with disease; DNED, Died, no evidence of disease; DOD, Died of disease; N/A, not available

Survival analysis was performed using the Kaplan-Meier method for disease-free survival (DFS) and overall survival (OS). Potential prognostic variables achieving significance level of 0.20 or less on univariate analysis were subsequently entered into a multivariable Cox-proportional hazards model and forward stepwise selection was used to determine the simplest model that best described the association in the data. Cox regression analysis included expression of the MCPyV LTA in the primary tumor and lymph metastases, staging and tumor localization. All statistical tests were 2-tailed, and a p-value of < 0.05 was considered significant.

## Results

### Demographics

In this current study 41 patients with Merkel cell carcinoma were included. The median age at first presentation was 76 years (range, 46–93 years), and the female/male sex ratio was 18/23. Twenty-one primary tumors were located in the head and neck (51%), whereas 10 on the extremities (24%) and 6 on the trunk (15%). In four patients no primary tumor was found (10%). At the time of first diagnosis 19 patients had stage I (46%), 6 patients stage II (15%), 10 patients stage III (24%) and 2 patients stage IV (5%) disease, respectively. Clinicopathologic features such as sex, age, staging and tumor localization are summarized in Table 1.

### Treatment

All patients underwent either open biopsy or primary wide local excision at first presentation for diagnostic tissue sampling. 18 patients (44%) were treated with tumor excision alone. 19 patients (46%) underwent postoperative radiotherapy and 2 patients (5%) received radiochemotherapy due to advanced primary disease. Lymph node samples were taken from sentinel node biopsies or neck dissections. 18 patients (44%) showed MCC positive lymph node involvement at the time of first diagnosis.

### Histopathology

In the entire cohort of 41 MCC patients, 54 specimens were retrieved for immunohistochemical staining for the MCPyV LTA. In total, 33 specimens (61%) were MCPyV-positive. Immunochemical staining was confirmed by PCR as described in material and methods. In the primary tumors 15 out of 32 were positive (47%)(Fig 1B) compared to 18 out of 22 lymph node metastases (82%)(Fig 1D). In primary specimens MCPyV expression was moderate in 5 cases and strong in 10 cases. In lymph node metastases 3 out of 18 positive specimens showed moderate expression. One nodal metastasis was virus-negative (Fig 1C) in a patient with virus-positive primary tumor. Four specimens from Merkel cell carcinoma of unknown primary (MCCUP) patients were MCPyV-positive (100%). Two primary tumors were virus-negative with virus-positive lymph node metastases (Fig 1D). In 5 primary tumors and 5 nodal metastases no virus staining was available. 61% of the female patients were MCPyV-positive compared to 65% of the male patients. The median age of virus-positive patients was 74 years (range 53–85) compared to 79 years (range 46–93) in virus-negative patients (Tables 1 and 2).

Cytokeratin 20 staining was positive in 96% (27/28), Chromogranin A in 83% (19/23) and NSE in 80% (12/15). Numbers represent specimens in which specific immunohistochemistry was performed.

### Outcomes

The median follow-up time was 44 months for all patients (range, 1–152 months). Recurrent disease was found in 21 patients (51%). At the time of last follow-up 13 patients were alive without disease, one patient was alive with disease, 12 patients had died of other disease and 15 patients had died from MCC. Out of these patients 11 were MCPyV-positive (73%) and only 4 were MCPyV-negative (27%)(Table 2).

**Fig 3:**
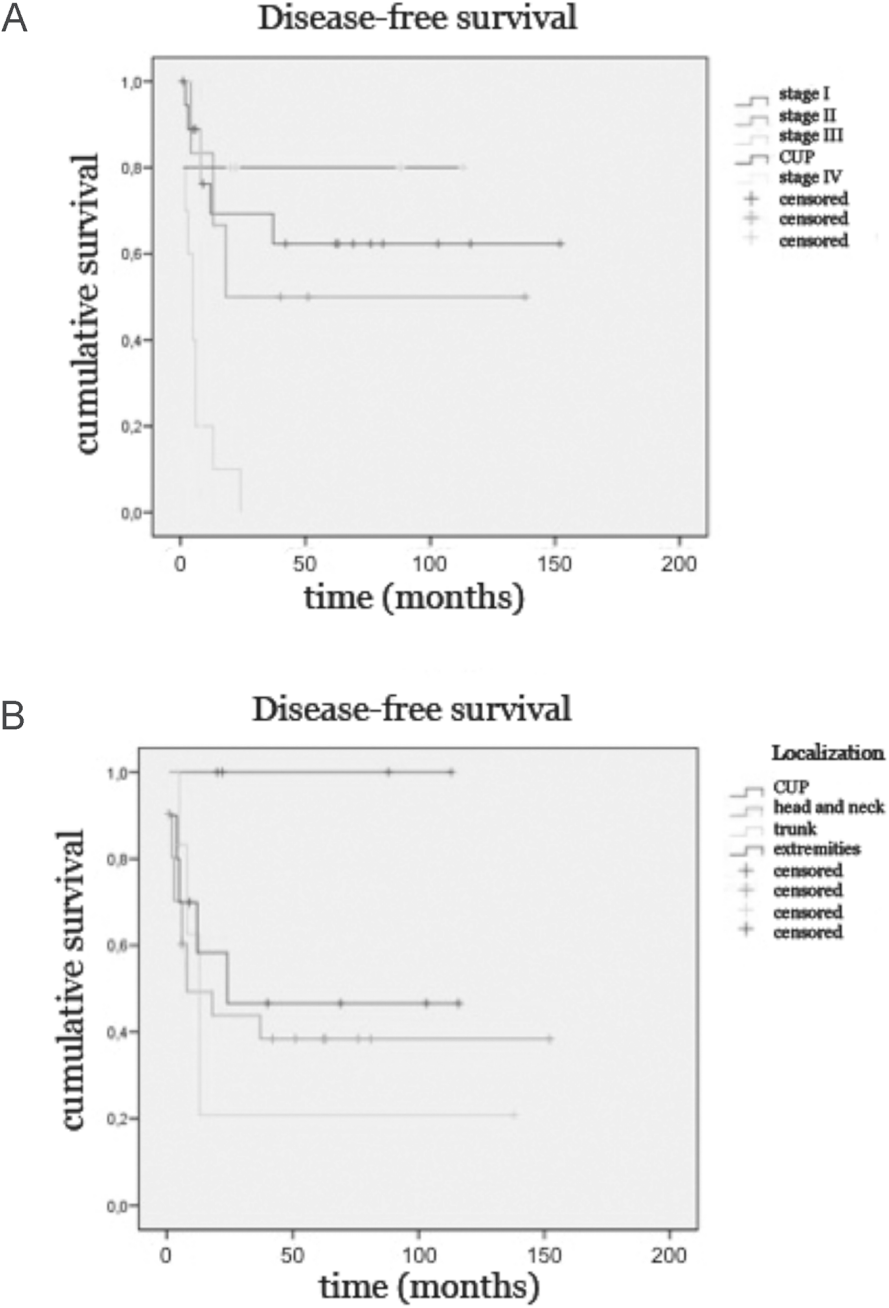
Kaplan-Meier survival curves depict disease-free survival of 41 MCC patients according to tumor stage (Fig 3A) and tumor localization (Fig 3B). Patients with higher tumor staging survived significantly shorter (p < 0.001) whereas patients with MCCUP had a better outcome than patients with primary tumors of the head and neck region, the trunk or the extremities (p = 0.268).

The 2-year DFS rates according to tumor stage were 70%, 50%, 10% and 0% for stage I, II, III and IV, respectively (Fig 3A). The 2-year OS rates were 72%, 68%, 30%, and 0% for stage I-IV, respectively. Of note patients with MCCUP showed better 2-years DFS (80%) and comparable OS (40%) rates to stage I disease (Fig 3A). Patient sex and age were no prognostic factors. Tumor stage was the most significant predictor of DFS (p = 0.001). Although not statistically significant, patients with MCCUP had better DFS (p = 0.268)(Fig 3B) and OS (p = 0.386) than patients with MCC of the head and neck, the trunk or the extremities.

**Fig 4:**
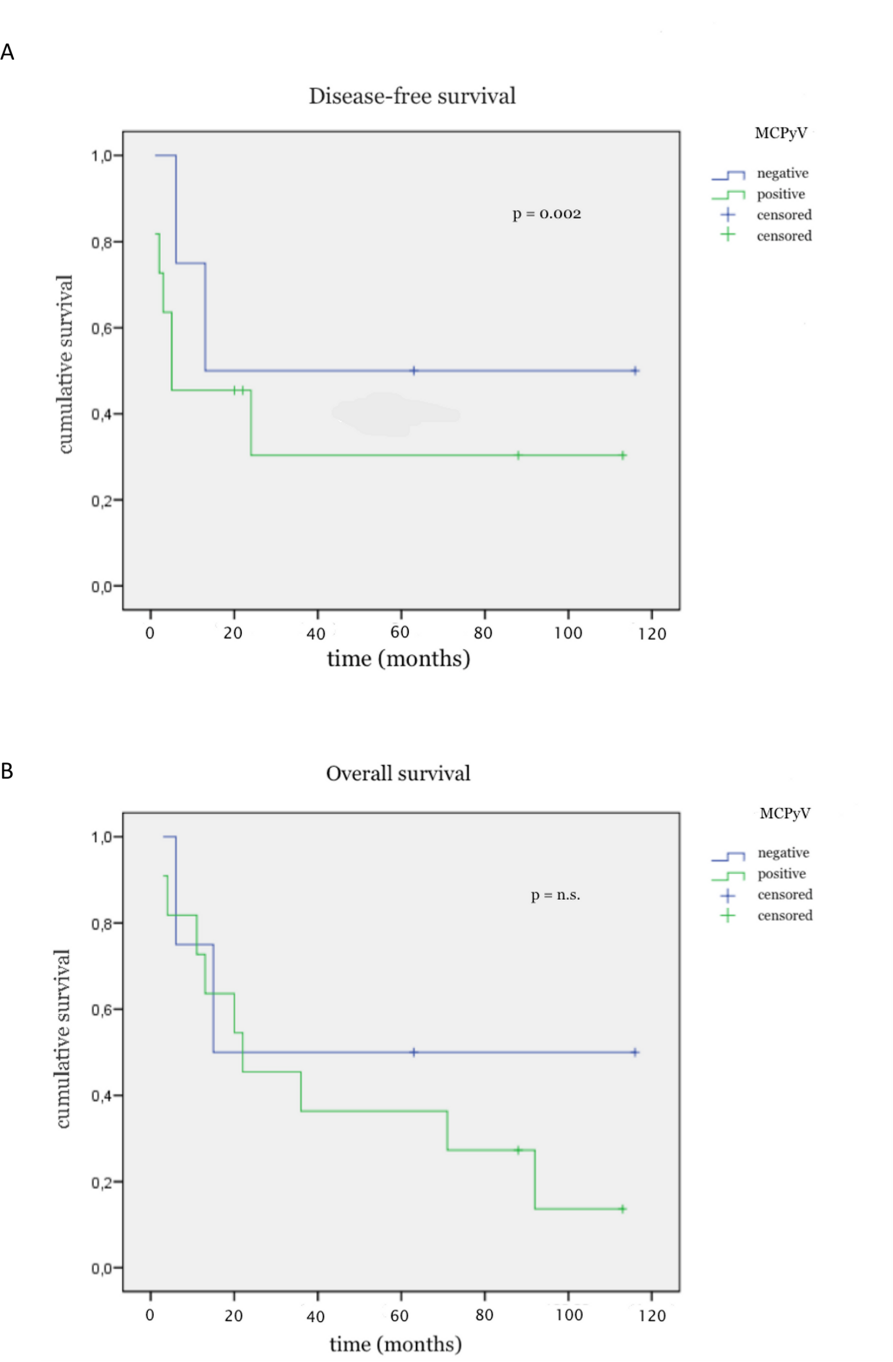
Disease-free survival (DFS) stratified by expression of the MCPyV large T-antigen (LTA) in lymph node metastases. Patients with LTA-positive (green line) MCC lymph node metastases had a shorter DFS (p = 0.002)(Fig 4A) and OS (p = n.s.)(Fig 4B) than patients with LTA-negative (blue line) lymph nodes. (n.s. = not significant).

In the entire cohort, MCPyV positive patients showed a higher recurrence rate than MCPyV negative patients (71% vs. 55%). Cox regression analysis including expression of the MCPyV LTA in the primary tumor and lymph metastases, staging and tumor localization showed that virus expression in the lymph nodes was a strong predictor for outcome. In particular, patients with MCPyV LTA positive lymph nodes had a worse DFS compared to patients with virus-negative lymph nodes (p = 0.002)(Fig 4A). Overall survival was not significantly linked to MCPyV expression (Fig 4B).

## Discussion

Since the first description of the human polyomvirus MCPyV and the development of MCC by Feng and colleagues in 2008 a series of studies have tried to identify the mechanism of DNA integration and tumor activation [4]. To date the current literature hypotheses that the viral infection occurs during early infancy via respiratory, cutaneous or fecal-oral routes [9,10]. Virus activation is triggered by aging, UV radiation, immunodeficiency due to HIV infection or immunosuppressive therapy. Thus, there might be two possible pathogenetic pathways of MCC in the human. Firstly, a virus induced oncogenesis in patients with a high viral load of the tumor and secondly, a MCC variant which is not viral associated but rather induced by UV radiation in sun-exposed areas of the skin [11,12]. The prognostic significance of MCPyV LTA in MCC primaries or lymph node metastates remains unclear. We therefore, conducted a retrospective analysis of MCC patients and correlated MCPyV LTA in lymph node metastes with clinical outcome.

The prevalence of MCPyV in the primary tumor of MCC patients varies between 43% in Australia, 69% in Northern America and 85–100% in Northern Europe [13–16]. The MCPyV can be detected either by immunohistochemistry using the CM2B4 mouse antibody or PCR. In our present study, the MCPyV LTA was detected by immunohistochemistry of paraffin embedded sections in 61% of the patients and confirmed by PCR. In particular, in the primary tumors 47% specimens were positive compared to 82% in lymph node metastases. Recent studies detected polyomavirus DNA positive lymph node metastases in 46% and 72% patients, respectively [14–16]. In accordance to this study, we observed a high MCPyV LTA expression in primary tumors as well as lymph node metastases. In one patient, the primary tumor was positive but the metastasis was found negative. In contrast, two primary tumors were virus-negative but showed virus-positive lymph node metastases. As for this finding we do not have an explanation, however, the primary tumor could have gone through a transformation after incorporation of the polyomavirus, which then could only be detected in the lymph node metastases.

Previous studies described a better survival of patients with polyomavirus positive MCC compared to patients which were virus negative. In particular, the OS of patients with MCPyV positive MCC was described as significantly higher than those with MCPyV negative MCC [17,18]. On the other hand, Loyo and colleagues found in their study on 25 patients no significant difference of the recurrence rate and OS in patients with histologically negative/MCPyV-positive lymph nodes compared to histologically negative/MCPyV-negative lymph nodes [19]. In our current study however, cox regression analysis including expression of the MCPyV LTA in the primary tumor and lymph metastases, staging and tumor localization showed that virus expression in lymph nodes was a predictor for poor outcome. In particular, patients with MCPyV LTA positive lymph nodes showed a significantly worse DFS compared to patients who lack MCPyV LTA expression. Moreover, 11 MCPyV-positive patients died from disease compared to only 4 MCPyV-negative patients. This finding is very interesting especially in regards to the fact that this group was slightly younger with a median age of 74 years compared to 79 years in the virus-negative group.

These results may strengthen the hypothesis that the virus in lymph node metastases leads to poorer prognosis [19]. An additional explanation might be given by distinct mechanisms of small T-antigen and large T-antigen induced carcinogenesis which are required for optimal MCC tumor growth [20]. However, the underlying cellular mechanisms, especially whether virus integration or replication are responsible for disease worsening, remain unknown. In out study, positive specimen showed mostly strong nuclear staining. Moderate protein expression was found in 5 primary tumors and 3 lymph node metastases. There was no difference in LTA expression levels in primary or metastatic disease.

Expectantly, tumor staging had the strongest influence on DFS in multivariate testing. The 2-year DFS rates according to tumor stage were 70%, 50%, 10% and 0% for stage I, II, III and IV, respectively. Patient age and sex were not found to be prognostic factors. However, the localization of the primary tumor resulted in a difference in patient outcome. In particular, patients with MCCUP showed a better DFS and OS compared to the head and neck, the trunk or the extremities. Patients with MCC of the head and neck showed worse survival rates compared to the trunk. This might be explained by the fact that the exposure to UV radiation is higher in this region and hence the polyomavirus plays a less important role in carcinogenesis. Indeed, in our patient cohort we observed only 43% MCPyV-positive primaries of the head and neck region compared to 50% MCPyV primaries of the trunk and 100% MCPyV-positive primaries of the extremities.

Despite the limited number of patients, the homogeneity of our study cohort emphasizes the importance of MCPyV as prognostic factor in areas of less sun exposure in MCC patients. MCC especially effects the elderly population with a high risk of early metastases and hence aggressive treatment is needed [21]. However, there might be a MCC patient group with worse outcome if lymph nodes are MCPyV positive and therefore, also need to be treated more aggressive than comparably staged patients with virus negative lymph nodes.

In conclusion, this is the first study to describe a worse outcome of patients with positive MCPyV expression. In particular, our results underline previous studies indicating poor outcome in patients with virus positive lymph node metastases. Standard diagnostic tests for MCPyV LTA might help to identify a subset of MCC patients with poor prognosis who could benefit from more aggressive treatment.

## References

[1] Toker C. Trabecular carcinoma of the skin. Arch Dermatol. 1972;105: 107–110. 5009611

[2] Erovic I, Erovic BM. Merkel cell carcinoma: The Past, the present, and the future. J Skin Cancer. 2013;2013: 929364 doi: 10.1155/2013/929364 23691324 10.1155/2013/929364 PMC3652192

[3] Deneve JL, Messina JL, Marzban SS, Gonzalez RJ, Walls BM, Fisher KJ, et al Merkel cell carcinoma of unknown primary origin. Ann Surg Oncol. 2012;19: 2360–2366. doi: 10.1245/s10434-011-2213-2 22271206 10.1245/s10434-011-2213-2 PMC4504007

[4] Frigerio B, Capella C, Eusebi V, Tenti P, Azzopardi JG. Merkel cell carcinoma of the skin: the structure and origin of normal Merkel cells. Histopathology. 1983;7: 229–249. 6852784 10.1111/j.1365-2559.1983.tb02238.x

[5] Feng H, Shuda M, Chang Y, Moore PS. Clonal integration of a polyomavirus in human Merkel cell carcinoma. Science. 2008;319: 1096–1100. doi: 10.1126/science.1152586 18202256 10.1126/science.1152586 PMC2740911

[6] Moens U, Rasheed K, Abdulsalam I, Sveinbjornsson B. The role of Merkel cell polyomavirus and other human polyomaviruses in emerging hallmarks of cancer. Viruses. 2015;7: 1871–1891. doi: 10.3390/v7041871 25866902 10.3390/v7041871 PMC4411681

[7] Nghiem P, Sober A, Lemos B. Merkel cell carcinoma In: Edge SB, Byrd DR, Compton CC, et al, editors. AJCC cancer staging manual. 7th ed New York, USA: Springer; 2010 p. 315–323.

[8] Haymerle G, Fochtmann A, Kunstfeld R, Pammer J, Erovic BM. Management of Merkel cell carcinoma of unknown primary origin: the Vienna Medical School experience. Eur Arch Otorhinolaryngol. 2015;272: 425–429. doi: 10.1007/s00405-014-2974-x 24633244 10.1007/s00405-014-2974-x

[9] Tolstov YL, Pastrana DV, Feng H, Becker JC, Jenkins FJ, Moschos S, et al Human Merkel cell polyomavirus infection II. MCV is a common human infection that can be detected by conformational capsid epitope immunoassays. Int J Cancer. 2009;125: 1250–1256. doi: 10.1002/ijc.24509 19499548 10.1002/ijc.24509 PMC2747737

[10] Chen T, Hedman L, Mattila PS, Jartti T, Ruuskanen O, Soderlund-Venermo M, et al Serological evidence of Merkel cell polyomavirus primary infections in childhood. J Clin Virol. 2011;50: 125–129. doi: 10.1016/j.jcv.2010.10.015 21094082 10.1016/j.jcv.2010.10.015

[11] Moll I, Bladt U, Jung EG. Presence of Merkel cells in sun-exposed and not sun-exposed skin: A quantitative study. Arch Dermatol Res. 1990;282: 213–216. 1695498 10.1007/BF00371638

[12] Popp S, Waltering S, Moll I, Boukamp P. UV-B-type mutations and chromosomal imbalances indicate common pathways for the development of Merkel and skin squamous cell carcinomas. Int J Cancer. 2002;99: 352–360. doi: 10.1002/ijc.10321 11992403 10.1002/ijc.10321

[13] Viscidi RP, Rollison DE, Sondak VK, Silver B, Messina JL, Giuliano AR, et al Age-specific seroprevalence of Merkel cell polyomavirus, BK virus, and JC virus. Clin Vaccine Immunol. 2011;18: 1737–1743. doi: 10.1128/CVI.05175-11 21880855 10.1128/CVI.05175-11 PMC3187023

[14] Garneski KM, Warcola AH, Feng Q, Kiviat NB, Leonard JH, Nghiem P. Merkel cell polyomavirus is more frequently present in North American than Australian Merkel cell carcinoma tumors. J Invest Dermatol. 2009;129: 246–248. doi: 10.1038/jid.2008.229 18650846 10.1038/jid.2008.229 PMC2605200

[15] Becker JC, Houben R, Ugurel S, Trefzer U, Pföhler C, Schrama D. MC polyomavirus is frequently present in Merkel cell carcinoma of European patients. J Invest Dermatol. 2009;129: 248–250. doi: 10.1038/jid.2008.198 18633441 10.1038/jid.2008.198

[16] Erovic BM, Al Habeeb A, Harris L, Goldstein DP, Ghazarian D, Irish JC. Significant overexpression of the Merkel cell polyomavirus (MCPyV) large T antigen in Merkel cell carcinoma. Head Neck. 2013;35: 184–189. doi: 10.1002/hed.22942 22307956 10.1002/hed.22942

[17] Kuwamoto S. Recent advances in the biology of Merkel cell carcinoma. Hum Pathol. 2011;42: 1063–1077. doi: 10.1016/j.humpath.2011.01.020 21641014 10.1016/j.humpath.2011.01.020

[18] Leroux-Kozal V, Lévêque N, Brodard V, Lesage C, Dudez O, Makeieff M, et al Merkel cell carcinoma: histopathologic and prognostic features according to the immunohistochemical expression of Merkel cell polyomavirus large T antigen correlated with viral load. Hum Pathol. 2015;46: 443–453. doi: 10.1016/j.humpath.2014.12.001 25623078 10.1016/j.humpath.2014.12.001

[19] Loyo M, Schussel J, Colantuoni E, Califano J, Brait M, Kang S et al Detection of Merkel cell virus and correlation with histologic presence of Merkel cell carcinoma in sentinel lymph nodes. Br J Cancer. 2012;106: 1314–1319. doi: 10.1038/bjc.2012.73 22415238 10.1038/bjc.2012.73 PMC3314790

[20] Spurgeon ME, Lamber PF. Merkel cell polyomavirus: A newly discovered human virus with oncogenic potential. Virology. 2013;435: 118–130. doi: 10.1016/j.virol.2012.09.029 23217622 10.1016/j.virol.2012.09.029 PMC3522868

[21] Schneider S, Thurnher D, Erovic BM. Merkel cell carcinoma: interdisciplinary management of a rare disease. J Skin Cancer. 2013;2013: 189342 doi: 10.1155/2013/189342 23401779 10.1155/2013/189342 PMC3557626

